# Effect of Solid-State Fermentation on Nutritional Quality of Leaf Flour of the Drumstick Tree (*Moringa oleifera* Lam.)

**DOI:** 10.3389/fbioe.2021.626628

**Published:** 2021-04-12

**Authors:** Honghui Shi, Endian Yang, Yun Li, Xiaoyang Chen, Junjie Zhang

**Affiliations:** ^1^State Key Laboratory for Conservation and Utilization of Subtropical Agro-Bioresources, South China Agricultural University, Guangzhou, China; ^2^Guangdong Key Laboratory for Innovative Development and Utilization of Forest Plant Germplasm, Guangzhou, China; ^3^Guangdong Province Research Center of Woody Forage Engineering Technology, Guangzhou, China; ^4^College of Forestry and Landscape Architecture, South China Agricultural University, Guangzhou, China; ^5^College of Biological Sciences and Technology, Beijing Forestry University, Beijing, China

**Keywords:** *Moringa oleifera* Lam., solid-state fermentation, nutritional quality, antioxidant activity, protein

## Abstract

The drumstick tree is a fast-growing multipurpose tree with a large biomass and high nutritional value. However, it has rarely been exploited as a protein source. This study investigated solid-state fermentation induced by *Aspergillus niger*, *Candida utilis* and *Bacillus subtilis* to obtain high-quality protein feed from drumstick leaf flour. The results showed that fermentation induced significant changes in the nutritional composition of drumstick leaf flour. The concentrations of crude protein, small peptides and amino acids increased significantly after fermentation. The protein profile was also affected by the fermentation process. Macromolecular proteins in drumstick leaf flour were degraded, whereas other high molecular weight proteins were increased. However, the concentrations of crude fat, fiber, total sugar and reducing sugar were decreased, as were the anti-nutritional factors tannins, phytic acid and glucosinolates. After 24 h fermentation, the concentrations of total phenolics and flavonoids were increased. The antioxidant capacity was also significantly enhanced.

## Introduction

The demand for animal protein for human nutrition is still rising in the developing world and the cost of feed for livestock is also increasing (Tufarelli et al., 2018). Therefore, a new source of feed protein is urgently needed to solve these problems. Among the available forage crops, special focus has been given to the effects of *Moringa oleifera* Lam. on livestock growth and production (Cui et al., 2018; Yusuf et al., 2018; Zeng et al., 2018). *Moringa oleifera* Lam., commonly known as the drumstick tree, belongs to the family of Moringaceae and is widely distributed in tropical and subtropical regions (Popoola and Obembe, 2013; Leone et al., 2015). It is extremely nutritious, containing high levels of protein, vitamins, minerals and phytochemicals (Leone et al., 2015). These nutritional traits together with its high production of leaf mass and adaptability to climate conditions and dry soils make drumstick a potential high quality feed source for livestock (He et al., 2020). Dietary inclusion of drumstick has been shown to enhance nutritional status, growth performance, milk production and meat quality in several livestock species (Cui et al., 2018; Kholif et al., 2018; Zeng et al., 2018). Such characteristics show that drumstick is a rich source of nutrients and biological activities for livestock, which could help to relieve the shortage of feed resources and decrease the need for antibiotics. However, anti-nutritional factors (ANF) in drumstick, such as tannins, phytic acid and glucosinolates, could affect the palatability, digestion and absorption, limiting its nutrition availability (Stevens et al., 2016). Moreover, most of the proteins are insoluble despite drumstick tree leaves having relatively high protein content (Teixeira et al., 2014). So far, the nutritional value of drumstick leaves has mainly been improved by heating, grinding, cooking and other physical and chemical means (Vongsak et al., 2013; Adedapo et al., 2015; Phatsimo et al., 2015). Although some of the ANF can be removed, the nutrient content may also be destroyed by these processes. Therefore, a more suitable process for improving drumstick feed quality is needed.

Solid-state fermentation (SSF) involves the growth of microorganisms on substrates with limited water content (Dulf et al., 2017). Numerous studies have demonstrated that the functionalities of various agricultural by-products can be enhanced by SSF. Indeed, many beneficial compounds have been produced through SSF, such as organic acids, enzymes, aromatic and flavor compounds, as well as bioactive compounds (Bennett and Yang, 2012; Dulf et al., 2016; Jin et al., 2016). In recent years, SSF has been widely used in the feedstock industry and has shown good prospects for promoting nutrient utilization and decreasing ANF levels (Chi and Cho, 2016; Wang et al., 2018a). Although, several studies have been conducted on the SSF of drumstick leaf flour (DLF), they mainly focused on the selection of strains, optimization of fermentation technology and evaluation of the nutritional value before and after fermentation (Zhang et al., 2017; Wang et al., 2018b). Only a few studies have examined the dynamic changes of nutrients and antioxidant components during fermentation, and even fewer have reported on the antioxidant activity of DLF after fermentation.

The type of microorganism used for inoculation affects the nutritional quality of the fermented feed. Due to the developed enzyme system, *Aspergillus Niger* and *Bacillus subtilis* are commonly used in ANF degradation and hydrolysis of macronutrient factors (Wang et al., 2020). *Candida utilis* is easy to be grown with other strains and often used for the production of single-cell protein for its high content of protein, vitamins B, amino acids and other nutrients (Xie et al., 2016). This study evaluated SSF processes induced by *A. niger*, *C. utilis* and *B. subtilis* for the mixed fermentation of drumstick leaves. Effects on the nutrient composition, physical and chemical properties and functional activity of drumstick leaves were studied. The main aims were to obtain a high nutrition, multi-functional feed, broaden the utilization ways of drumstick feed and provide some theoretical basis for the further processing of drumstick resources.

## Materials and Methods

### Microorganisms

*A. niger* strain GIM 3.576 and *C. utilis* strain GIM 1.427 were obtained from the Guangdong Microbial Strain Preservation Center. *B. subtilis* CICC 31188 was obtained from the China Center of Industrial Culture Collection (CICC). GIM 3.576 was cultured on potato dextrose agar (PDA) slants for 3 days at 28°C, GIM 1.427 was cultured on yeast extract peptone dextrose (YPD) slants for 3 days at 28°C and CICC 31188 was cultured on Luria-Bertani (LB) slants for 2 days at 30°C. After being activated for 3 generations, GIM 3.576 was cultured in PDA solid medium until mycelia had spread over the entire petri dish. The spores were then rinsed with sterile saline solution, and the concentration of the spore suspension was calculated and diluted to 1 × 10^7^ spores/mL on a blood cell counting board. GIM 1.427 was incubated in YPD liquid medium at 28°C and 150 r/min for 3 days then diluted to a concentration of 1 × 10^8^ spores/mL. CICC 31188 was cultured in LB liquid medium at 30°C and 250 r/min and diluted to a concentration of about 1 × 10^8^ spores/mL.

### Solid-State Fermentation

Drumstick leaf samples were harvested from trees of the PKM-1 cultivar. After harvesting, the samples were sun-dried until a constant weight was reached, then ground to a powder and passed through a 40-mesh sieve. Portions of 50 g were packed and sealed in polypropylene bags for sterilization in an autoclave at 121°C for 20 min. After cooling, sterilized water was added to adjust the initial water content to 50%. Then 6% (v/w) of the GIM 3.576 spore suspension was inoculated onto each DLF. After fermentation for 24 h, 6% (v/w) of the GIM 1.427 suspension and 12% (v/w) of the CICC 31188 suspension were inoculated simultaneously and fermented in a biochemical incubator at 32°C for a total of 7 days (Shi et al., 2020). Some of the fermentation samples were stored at −20°C for detection of the active substances, whereas the rest were dried at 50°C, crushed and screened for chemical analysis.

### Scanning Electron Microscopy

Samples were prepared by a standard procedure of alcohol dehydration for scanning electron microscopy (SEM, Carl Zeiss, EVO MA15, Germany) analysis. After dehydration, the samples were placed on an aluminum column and sprayed with 12 nm of gold using ion sputtering coater (Leica, EM ACE 600, Germany). SEM images were recorded at 25 kV and with high vacuum modes.

### Protein and Amino Acid Analysis

The crude protein (CP) concentration was analyzed using methods of the Association of Official Analytical Chemists (AOAC, 2005). The protein profile was analyzed by sodium dodecyl sulfate-polyacrylamide gel electrophoresis (SDS-PAGE) according to the method described by Zuo et al. (2017) with slight modifications. Proteins from unfermented and fermented samples were extracted using the plant total protein extraction kit (KeyGen Biotech, China), denatured with 2 × Tris-glycine SDS loading buffer and separated using a 12% gel for SDS-PAGE at 110 V. The gel was stained with Coomassie Brilliant Blue R250 and de-stained with 8% acetic acid until the protein bands were visible. The amino acid (AA) profile was analyzed using an automatic AA analyzer (L-8900, Hitachi, Tokyo, Japan). Trichloroacetic acid-soluble protein (TCA-SP) was analyzed as reported by Ovissipour et al. (2009). Small peptide concentration was calculated by subtracting the concentration of free amino acids from that of TCA-SP.

### Chemical Analysis

Dry matter (DM), ether extract (EE), crude fiber (CF), crude ash (CA), calcium (Ca) and phosphorus (P) were analyzed using methods of the Association of Official Analytical Chemists (AOAC, 2005). Neutral detergent fiber (NDF), acid detergent fiber (ADF) and lignin were determined by methods described by Van Soest et al. (1991). Potassium (K), sodium (Na), magnesium (Mg), copper (Cu), zinc (Zn) and iron (Fe) were determined by atomic absorption spectrophotometry as described by the AOAC (2005). Total sugars and reducing sugars were determined by 3,5-dinitrosalicylic acid colorimetry (Miller, 1959). Phytic acid was determined according to the method described by Gao et al. (2007). Glucosinolates were determined by palladium chloride colorimetry as described by Hu et al. (2010).

### Determination of Total Phenolic, Tannin and Flavonoid Content

A methanol extract from each sample powder was prepared according to the method described by Zuo and Chen (2002). The lyophilized samples were extracted twice with 80% (v/v) methanol for 3 h at room temperature and filtered through a 0.45 μm injection filter. The total phenolic concentration of the methanol-extracted samples was determined using the Folin-Ciocalteu procedure and expressed as grams of gallic acid equivalents (GAE) in 100 g of the extract (Generalić Mekinić et al., 2014). The concentration of tannins was calculated as the difference between the concentrations of total phenols and simple phenols after removing tannin from the extract using insoluble polyvinylpyrrolidone. The total flavonoid concentration was analyzed using the aluminum chloride colorimetric method and expressed as grams of rutin equivalents (RUE) in 100 g of the extract (Lin and Tang, 2007).

### Determination of Antioxidant Capacity

The ABTS^+^ (2,2′-azino-bis (3-ethylbenzothiazoline6-sulfonic acid) radical cation) radical-scavenging capacity of methanol extracts of samples were evaluated according to the method of Sasipriya and Siddhuraju (2012) with slight modifications. The stock solution was prepared by reacting 5 mL of 7 mM ABTS with 88 μL of 40 mM potassium persulfate and allowing the mixture to stand in the dark at room temperature for 12–16 h before use. The stock solution was diluted with methanol to give an absorbance of about 0.700 ± 0.020 at 734 nm. To determine the antioxidant capacity, 0.5 mL of methanolic extract samples (final concentration 1 mg/mL) and 4.0 mL of stock solution were mixed and incubated for 6 min. The absorbance was monitored at 734 nm.

The DPPH (2,2-diphenil-1-picrylhydrazyl) radical-scavenging capacity of methanol extracts of samples was evaluated according to the method of Arora and Chandra (2010) with slight modifications. A 100 μM solution of DPPH was prepared in absolute methanol, and 2.0 mL of the DPPH solution was added to 0.5 mL of a methanolic extract of a sample (final concentration 1 mg/mL). After thorough mixing, the solution was kept in the dark at room temperature for 30 min. The absorbance of the samples was measured using an UV-visible spectrophotometer at 517 nm against a methanol blank. Each sample was tested three times and the values were averaged.

The free radical scavenging activity (RSA) was calculated as a percentage using the following equation:

RSA(%)=[1-(Ai-Aj)/Ae]×100%

where Ae is the absorbance of the control, Ai is the absorbance of the sample extract and Aj is the absorbance of the control of sample.

The total antioxidant capacity was determined using the ferric reducing ability of plasma antioxidant power (FRAP) assay. The procedure was conducted according to the manufacturer’s instruction for the T-AOC kit (Jiancheng Bioengineering Institute, Nanjing, China).

### Statistical Analysis

All processing treatments were performed in triplicate. The statistical analysis was carried out using SPSS 23.0 software (SPSS Inc., Chicago, IL, United States), Duncan’s multiple range test was used to detect differences among means. A *p*-value <0.05 was considered significant. Pearson’s correlation analysis was also performed.

## Results and Discussion

### Microscopic Observation

**FIGURE 1:**
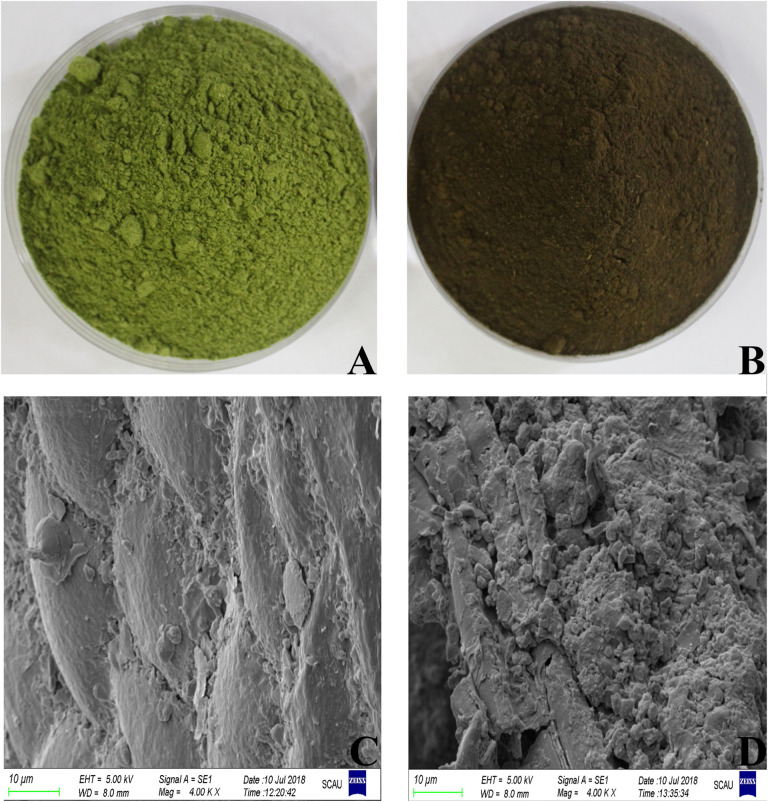
Phenotype and scanning electron microscope images (×4,000) of UDLF **(A,C)** and FDLF **(B,D)**.

During the fermentation process, the color of DLF changed from green to dark brown(Figure 1)accompanied by a sweet smell. The degradation of organic matter produces water, CO_2_, and heat during aerobic fermentation. High temperature will accelerate the degradation of chlorophyll and cause caramelization and Maillard reaction, producing Browning. This phenomenon was also observed in the fermentation of lupin flour (Olukomaiya et al., 2020). The microstructure was clearly different between fermented DLF (FDLF) and unfermented DLF (UDLF) (Figure 1). An irregularly shaped and rough surface was mainly observed in FDLF, whereas the microstructure of DLF was more regular and the surface smooth. This change of microstructure may increase the surface area of the substrate and facilitate the full reaction between enzyme and substrate.

### Effect of Solid-State Fermentation on Proteins and Amino Acids

The protein content is the most important parameter that determines the overall quality of animal feed products. It is well known that the protein content can vary depending on the microorganism used and their carbon and nutrient accessibility, as well as the cultivation conditions, such as carbon and nutrient sources, water content and pH. The analysis showed that the average concentration of CP in the raw flour was 28.42% (Table 1), close to the figure reported by Teixeira et al. (2014) but lower than the values reported by Moyo and Masika (2011), most likely due to differences in the growth stage and planting conditions (Mendieta-Araica et al., 2013). SSF significantly increased the CP concentrations from 28.42 to 40.98% (Table 1). The enrichment of CP could be the result of increased fungal biomass, suggesting that the treated substrate could act as a good protein source for livestock. However, it could also be due to the concentration effect caused by the aggravation of dry matter loss.

**TABLE 1:** Nutrient composition of FDLF and UDLF.

Item	UDLF	FDLF
CP,%	28.42 ± 1.17^b^	40.98 ± 0.59^a^
Small peptides,%	5.72 ± 0.20^b^	12.03 ± 0.12^a^
EE,%	5.43 ± 0.18^a^	3.75 ± 0.15^b^
CF,%	7.25 ± 0.02^a^	2.17 ± 0.04^b^
Ash,%	10.49 ± 0.03^b^	16.75 ± 0.02^a^
Total sugars,%	18.49 ± 0.62^a^	5.34 ± 0.88^b^
Reducing sugars,%	12.38 ± 0.81^a^	1.69 ± 0.16^b^
NDF,%	24.41 ± 2.11^b^	38.61 ± 2.97^a^
ADF,%	13.40 ± 2.06	12.97 ± 2.75
Lignin,%	1.30 ± 0.16^b^	2.13 ± 0.38^a^
Hemicellulose,%	11.01 ± 0.29^b^	22.40 ± 1.68^a^
Cellulose,%	12.62 ± 1.49^a^	7.50 ± 0.84^b^
Ca,%	2.34 ± 0.01^b^	3.64 ± 0.06^a^
P,%	0.25 ± 0.00^b^	0.42 ± 0.01^a^
K,%	1.04 ± 0.02^b^	1.28 ± 0.01^a^
Na,%	0.03 ± 0.01^b^	0.12 ± 0.01^a^
Mg,%	0.96 ± 0.02^b^	1.31 ± 0.02^a^
Fe, mg/100 g	41.70 ± 0.57^b^	73.05 ± 1.06^a^
Cu, mg/100 g	1.25 ± 0.01^b^	1.81 ± 0.01^a^
Zn, mg/100 g	2.45 ± 0.09^b^	3.53 ± 0.06^a^
Tannins, mg/g	14.60 ± 0.58^a^	8.59 ± 0.45^b^
Phytic acid, mg/g	18.31 ± 0.71^a^	7.30 ± 0.74^b^
Glucosinolates, mg/g	19.11 ± 0.13^a^	14.72 ± 0.24^b^

*Means followed by the different letters in the same line are significantly different from each other at P ≤ 0.05 level, according to Duncan’s multiple range test.*

**FIGURE 2:**
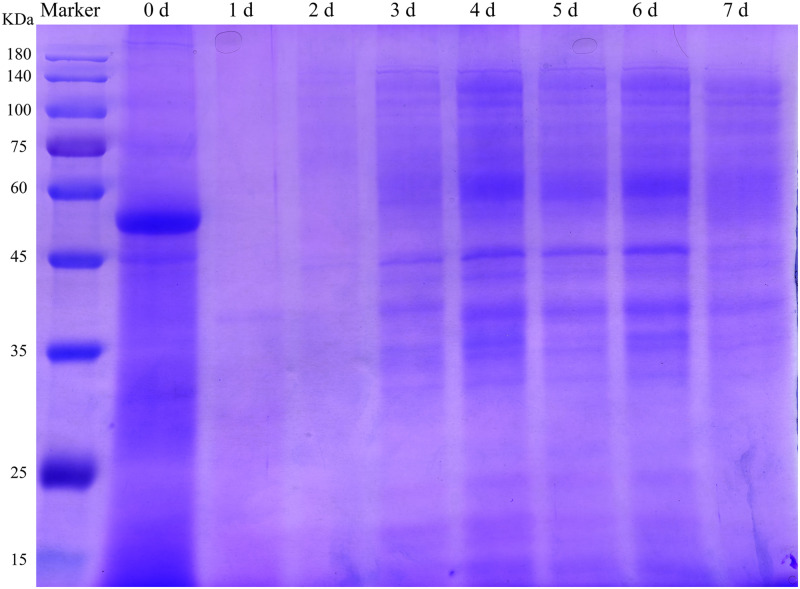
SDS-PAGE analysis of protein profiles in DLF during fermentation.

To analyze the influence of fermentation on the DLF protein profile, SDS-PAGE was performed. SSF affected the characteristics of proteins in DLF. The molecular weight of the main protein fractions in the unfermented DLF was 55 kDa (Figure 2). The maximal degradation of large proteins in the FDLF was almost complete after 24 h of fermentation. This was likely because highly active proteases secreted by the microorganisms during fermentation were able to decompose the large proteins (Chi and Cho, 2016). This reduction of protein sizes is important to increase the digestibility of protein. However, from the third day of fermentation, the color of the protein bands gradually deepened and bands appeared at around 33–45 kDa and 60–140 kDa, similar to the results reported by Zuo et al. (2017). This could be due to the production of single-celled proteins in the late fermentation period. This phenomenon indicated that fermentation not only degraded the macromolecular proteins in DLF but also increased the abundance of other proteins with high molecular weight.

Drumstick leaves contain all essential amino acids, but the content varies greatly depending on growth environments, cultivation mode, tree age, leaf maturity and other conditions (Olaofe et al., 2013; Ndubuaku et al., 2014). Evaluation of the amino acid profile confirmed significant differences between the fermented substrate and raw flour (Table 2). The levels of most amino acids increased, whereas glutamic acid and lysine decreased. The alterations in amino acid profiles may vary depending on the microorganism used (Medeiros et al., 2018). The inoculated microorganisms may use glutamic acid and lysine for metabolic activity, resulting in a reduction in their concentrations in the fermented substrate compared to those in raw flour. After fermentation, the total amino acid content of DLF was 24.88%, which was significantly higher than that of the unfermented substrate. This may be due to enzymatic hydrolysis of large proteins into small amino acids or the microbial synthesis of some amino acids. The concentration of essential amino acids and non-essential amino acids in the fermented substrate also increased, by 1.81 and 1.76%, respectively. Animals not only have dietary requirements for essential amino acids but also need nutritionally non-essential amino acids to achieve maximum growth and production performance. Therefore, increasing non-essential amino acids in animal feeds is beneficial. It has been reported that peptides are more rapidly utilized than proteins and amino acids (Kodera et al., 2006). The small peptide concentration in our study increased from 5.72 to 12.03% after fermentation. The increased peptide content may positively affect the bioactivity of DLF because they may contribute to antioxidative and metal-chelating activities (Chi and Cho, 2016).

**TABLE 2:** Amino acid composition of FDLF and UDLF.

Amino acid composition,%	UDLF	FDLF
Arg	1.42 ± 0.02^b^	1.60 ± 0.01^a^
His	0.50 ± 0.02^b^	0.58 ± 0.01^a^
Ile	0.97 ± 0.06^b^	1.15 ± 0.03^a^
Leu	2.06 ± 0.05^b^	2.38 ± 0.04^a^
Lys	1.40 ± 0.10	1.35 ± 0.02
Phe	1.31 ± 0.06^b^	1.53 ± 0.01^a^
Thr	1.12 ± 0.05^b^	1.38 ± 0.02^a^
Val	1.23 ± 0.08^b^	1.47 ± 0.01^a^
Trp	0.65 ± 0.07^b^	1.03 ± 0.11^a^
Ala	1.49 ± 0.06^b^	1.80 ± 0.02^a^
Asp	2.13 ± 0.11^b^	2.48 ± 0.10^a^
Cys	0.11 ± 0.04^b^	0.15 ± 0.02^a^
Glu	2.80 ± 0.08	2.69 ± 0.20
Gly	1.21 ± 0.01^b^	1.55 ± 0.05^a^
Pro	1.01 ± 0.04^b^	1.44 ± 0.01^a^
Ser	1.07 ± 0.04^b^	1.27 ± 0.04^a^
Tyr	0.84 ± 0.01^b^	1.03 ± 0.01^a^
Essential AA,%	10.66 ± 0.51^b^	12.47 ± 0.26^a^
Non-essential AA,%	10.65 ± 0.24^b^	12.41 ± 0.20^a^
Total AA	21.31 ± 0.75^b^	24.88 ± 0.46^a^

*Means followed by the different letters in the same line are significantly different from each other at P ≤ 0.05 level, according to Duncan’s multiple range test.*

### Effect of Solid-State Fermentation on the Chemical Composition

The results regarding the chemical composition of the fermented substrate and raw flour are shown in Table 1. SSF increased the content of crude ash, neutral washing fiber and lignin. In addition, the CF concentration of the fermented substrate was decreased markedly, by 70.07%, compared to the control. There were also clear reductions in total sugars and reducing sugars from 18.49 and 12.38% to 5.34 and 1.69%, respectively (Table 1). Meanwhile, the ether extract (fat) concentration decreased by 30.94% compared with that before fermentation. This suggests that the microbial fermentation process consumed carbohydrates and fats, especially small molecules of sugars. At the same time, SSF resulted in an increase in the mineral content, probably due to the metabolic activity of the microorganisms or dry matter loss.

### Effect of Solid-State Fermentation on Total Flavonoid, Phenolic Content and Antioxidant Capacity

**FIGURE 3:**
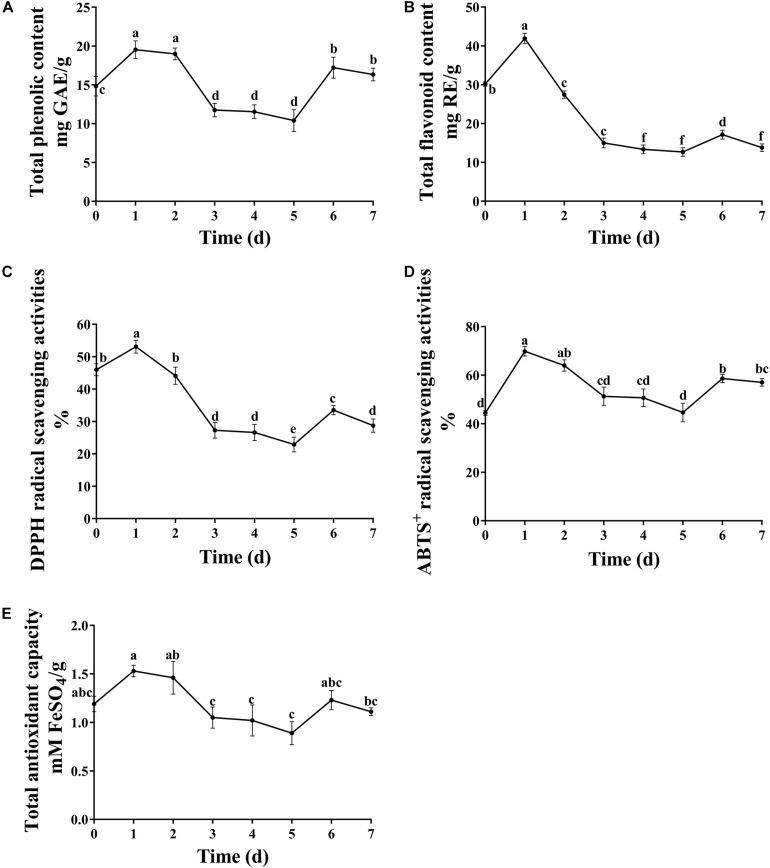
Dynamic changes in total phenolic, flavonoid content and antioxidant capacity. **(A)** Total phenolic content, **(B)** total flavonoid content, **(C)** DPPH radical scavenging activities, **(D)** ABTS^+^ radical scavenging activities, and **(E)** total antioxidant capacity. Different letters indicate significantly difference at *p* ≤ 0.05 level.

Antioxidants are free radical scavengers that can protect the body from free radicals that can cause a variety of diseases, including ischemia, asthma, anemia, dementia and arthritis. Phenols and flavonoids are considered to be some of the safest natural antioxidants, and fermentation technology is an effective way to increase the concentration of these compounds. In this study, the concentration of total phenols and flavones reached the maximum value on the first day of fermentation. Afterward, the content of total phenols and flavonoids decreased as the fermentation continued (Figure 3). The proposed reason for the increased content was that various extracellular enzymes such as cellulase, pectinase, xylanase and so on, secreted by microorganisms destroy the intact cell wall structure of plants, releasing flavonoids from within cells and phenols bound to the cell walls during fermentation (Dey et al., 2016). *A. niger* used in the present study is a microorganism which has been found in many SSF studies to liberate the phenolic compounds, such as plum fruit by-products (Dulf et al., 2016), apricot pomace (Dulf et al., 2017). However, prolonged fermentation may lead to the diffusion and oxidation of phenolic substances.

It is crucial to evaluate the antioxidant potential of extracts using more than one method due to the different mechanisms of antioxidant activity. In the present study, the antioxidant activity of fermented substrates was measured using ABTS^+^, DPPH free radical scavenging methods and FRAP. The antioxidant activity showed a trend of first increasing and then decreasing with increasing fermentation time (Figure 3), similar to results obtained with okra seeds during fermentation by Adetuyi and Ibrahim (2014). The antioxidant activity of DLF after fermentation was slightly lower than that before fermentation. Hossain et al. (2017) suggested that this might be because of a too long fermentation time and reduced content of phenols and other substances. Many studies have shown that the antioxidant capacity of plants is directly related to the content of phenolic compounds and flavones. In this study, Pearson’s correlation analysis showed that flavonoids were only slightly positively correlated with DPPH radical scavenging rate and total antioxidant capacity, whereas total phenols were highly positively correlated with the three antioxidant indexes tested (Table 3), indicating that the total phenols and flavonoid concentrations of FDLF were closely related to antioxidant activity. However, total phenols and flavonoids are not the only factors affecting antioxidant activity. Small peptides and amino acids, such as leucine, methionine, tyrosine, histidine, and tryptophan, can also make the fermented sample more reductive (Sarmadi and Ismail, 2010; Sanjukat et al., 2015). In addition, determination of phenolic substances with the Folin-Ciocalteu reagent may be influenced by a variety of non-phenolic compounds, such as reducing sugars, aromatic amino acids and citric acids, which often do not have free radical scavenging capacity. In general, the synergistic effect between phenols and other components in the solid fermentation of DLF may be the main reason for this phenomenon.

**TABLE 3:** Correlation analysis of the concentrations of total phenols and flavones and antioxidant activity in FDLF.

Item	DPPH free radical scavenging rate	ABTS^+^ free radical scavenging rate	Total antioxidant ability
total phenols	0.790*	0.856**	0.932**
flavones	0.975**	0.571	0.855**

*Means significant correlation at P ≤ 0.05 level and ** means highly significantly correlation at P ≤ 0.01 level.

### Effect of Solid-State Fermentation on Anti-nutritional Factors

Tannins, phytic acid and glucosinolates are the main anti-nutrients in DLF (Amaglo et al., 2010; Ogbe and Affiku, 2011; Chodur et al., 2018). Tannins can precipitate proteins, amino acids, alkaloids and other organic molecules in aqueous solution, which hinder the absorption of some nutrients due to complexation. Further, the bitter taste of tannins may affect the palatability of feed. High concentrations of tannins have an adverse effect on animal productivity and digestibility. Phytic acid is an organic acid that cannot be digested by animals with a single stomach. Therefore, phytic acid is eventually expelled in an animal’s feces, which may be degraded by aquatic microorganisms after entering water, releasing phosphorus and resulting in serious eutrophication of water (Singh and Satyanarayana, 2011). Although glucosinolates are non-toxic, they may decompose into glucose, isothiocyanate, nitrile, thiocarcinate and other toxic compounds through endogenous myrosinase. Their main anti-nutritional effects include reducing the palatability of feed, inducing iodine deficiency and damaging liver and kidney function. They are also more harmful to non-ruminants than ruminants (Tripathi and Mishra, 2007). Nevertheless, phytase, tannase and other enzymes can degrade these substances. *Aspergillus* is considered to be the main source of these enzymes and is known to be capable of degrading tannins, phytic acid and glucosinolates (Shi et al., 2016). In this study, the phytic acid and tannin concentrations of DFL were 18.31 and 14.60 mg/g, respectively, which are close to the values of 22.3 and 16.3 mg/g reported by Stevens et al. (2016). After fermentation, the levels of tannins, phytic acid and glucosinolates were drastically decreased (Table 1). This might have been caused by the secretion of tannase, phytase and other biological enzymes, which are able to break down tannins, phytic acid and glucosinolates.

## Conclusion

In this study, SSF by *A. niger*, *C. utilis* and *B. subtilis* was shown to be beneficial for improving the nutritional value of DLF. It not only increased the content of nutrients, such as CPs and small peptides, but also decreased the content of anti-nutrient factors, such as phytic acid and tannins. Our results suggest that the SSF method offers an effective approach for improving the quality of unconventional proteins sources such as DLF.

## Data Availability Statement

The raw data supporting the conclusions of this article will be made available by the authors, without undue reservation.

## Author Contributions

XC and JZ conceived and designed the research, wrote the manuscript, and obtained fundings. HS conducted the experiments. HS, EY, and YL collected and analyzed the data. All authors contributed to the article and approved the submitted version.

## Conflict of Interest

The authors declare that the research was conducted in the absence of any commercial or financial relationships that could be construed as a potential conflict of interest.

## References

[B1] Adedapo A. A. Falayi O. O. Oyagbemi A. A. (2015). Evaluation of the analgesic, antiinflammatory, antioxidant, phytochemical and toxicological properties of the methanolic leaf extract of commercially processed *Moringa oleifera* in some laboratory animals. *J. Basic Clin. Physiol. Pharmacol.* 26 491–499. 26020553 10.1515/jbcpp-2014-0105

[B2] Adetuyi F. O. Ibrahim T. A. (2014). Effect of fermentation time on the phenolic, flavonoid and vitamin C contents and antioxidant activities of okra (*Abelmoschus esculentus*) seeds. *Nigerian Food J.* 32 128–137. 10.1016/s0189-7241(15)30128-4

[B3] Amaglo N. K. Bennett R. N. Lo Curto R. B. Rosa E. S. Turco V. L. (2010). Profiling selected phytochemicals and nutrients in different tissues of the multipurpose tree *Moringa oleifera* L., grown in Ghana. *Food Chem.* 122 1047–1054. 10.1016/j.foodchem.2010.03.073

[B4] AOAC (2005). *Official Methods of Analysis of the AOAC International.* Gaithersburg, MD: Association of Official Analytical Chemists International.

[B5] Arora D. S. Chandra P. (2010). Assay of antioxidant potential of two *Aspergillus* isolates by different methods under various physio-chemical conditions. *Braz. J. Microbiol.* 41 765–777. 10.1590/s1517-83822010000300029 24031554 PMC3768632

[B6] Bennett P. Yang S. T. (2012). Beneficial effect of protracted sterilization of lentils on phytase production by *Aspergillus ficuum* in solid state fermentation. *Biotechnol. Prog.* 28 1263–1270. 10.1002/btpr.1603 22848026

[B7] Chi C. H. Cho S. J. (2016). Improvement of bioactivity of soybean meal by solid-state fermentation with *Bacillus amyloliquefaciens* versus *Lactobacillus spp*. and *Saccharomyces cerevisiae*. *J. LWT - Food Sci. Technol.* 68 619–625. 10.1016/j.lwt.2015.12.002

[B8] Chodur G. M. Olson M. E. Wade K. L. Stephenson K. K. Nouman W. Garima (2018). Wild and domesticated *Moringa oleifera* differ in taste, glucosinolate composition, and antioxidant potential, but not myrosinase activity or protein content. *Sci. Rep.* 8 7910–7995. 29789671 10.1038/s41598-018-26059-3 PMC5964143

[B9] Cui Y. M. Wang J. Lu W. Zhang H. J. Wu S. G. (2018). Effect of dietary supplementation with *Moringa oleifera* leaf on performance, meat quality, and oxidative stability of meat in broilers. *J. Poult. Sci.* 97 2836–2844. 10.3382/ps/pey122 29660045

[B10] Dey T. B. Chakraborty S. Jain K. K. Sharma A. (2016). Antioxidant phenolics and their microbial production by submerged and solid state fermentation process: a review. *Trends Food Sci. Technol.* 53 60–74. 10.1016/j.tifs.2016.04.007

[B11] Dulf F. V. Vodnar D. C. Dulf E. H. Pintea A. (2017). Phenolic compounds, flavonoids, lipids and antioxidant potential of apricot (*Prunus armeniaca* L.) pomace fermented by two filamentous fungal strains in solid state system. *Chem. Cent. J.* 11:92. 10.1186/s13065-017-0323-z PMC5608653 29086904

[B12] Dulf F. V. Vodnar D. C. Socaciu C. (2016). Effects of solid-state fermentation with two filamentous fungi on the total phenolic contents, flavonoids, antioxidant activities and lipid fractions of plum fruit (*Prunus domestica* L.) by-products. *Food Chem.* 209 27–36. 10.1016/j.foodchem.2016.04.016 27173530

[B13] Gao Y. Shang C. Maroof M. A. S. Biyashev R. M. Buss G. R. (2007). A modified colorimetric method for phytic acid analysis in soybean. *Crop Sci.* 47 1797–1803. 10.2135/cropsci2007.03.0122

[B14] Generalić Mekinić I. Skroza D. Ljubenkov I. Šimat V. Smole Možina S. Katalinić V. (2014). In vitro antioxidant and antibacterial activity of Lamiaceae phenolic extracts: a correlation study. *Food Technol. Biotechnol.* 52 119–127.

[B15] He L. Lv H. Chen N. Wang C. Zhang Q. (2020). Improving fermentation, protein preservation and antioxidant activity of *Moringa oleifera* leaves silage with gallic acid and tannin acid. *Bioresour. Technol.* 297:122390. 10.1016/j.biortech.2019.122390 31740244

[B16] Hossain A. Khatun A. Munshi M. K. Hussain M. S. Huque R. (2017). Study on antibacterial and antioxidant activities of raw and fermented *Moringa oleifera* Lam. leaves. *J. Microbiol. Biotechnol. Res.* 6 23–29.

[B17] Hu Y. Liang H. Yuan Q. Hong Y. (2010). Determination of glucosinolates in 19 Chinese medicinal plants with spectrophotometry and high-pressure liquid chromatography. *Nat. Prod. Res.* 24 1195–1205. 10.1080/14786410902975681 20645206

[B18] Jin B. Zepf F. Bai Z. Gao B. Zhu N. (2016). A biotech-systematic approach to select fungi for bioconversion of winery biomass wastes to nutrient-rich feed. *Process Saf. Environ. Prot.* 103 60–68. 10.1016/j.psep.2016.06.034

[B19] Kholif A. E. Gouda G. A. Olafadehan O. A. Abdo M. M. (2018). Effects of replacement of *Moringa oleifera* for berseem clover in the diets of *Nubian goats* on feed utilisation, and milk yield, composition and fatty acid profile. *Animal* 12 964–972. 10.1017/s1751731117002336 28988560

[B20] Kodera T. Hara H. Nishimori Y. Nio N. (2006). Amino acid absorption in portal blood after duodenal infusions of a soy protein hydrolysate prepared by a novel soybean protease D3. *J. Food Sci.* 71 S517–S525.

[B21] Leone A. Spada A. Battezzati A. Schiraldi A. Aristil J. Bertoli S. (2015). Cultivation, genetic, ethnopharmacology, phytochemistry and pharmacology of *Moringa oleifera* leaves: an overview. *Int. J. Mol. Sci.* 16 12791–12835. 10.3390/ijms160612791 26057747 PMC4490473

[B22] Lin J. Tang C. (2007). Determination of total phenolic and flavonoid contents in selected fruits and vegetables, as well as their stimulatory effects on mouse splenocyte proliferation. *Food Chem.* 101 140–147. 10.1016/j.foodchem.2006.01.014

[B23] Medeiros S. Xie J. Dyce P. W. Cai H. Y. Delange K. Zhang H. (2018). Isolation of bacteria from fermented food and grass carp intestine and their efficiencies in improving nutrient value of soybean meal in solid state fermentation. *J. Anim. Sci. Biotechno.* 9:29. 10.1186/s40104-018-0245-1 PMC5885361 29632666

[B24] Mendieta-Araica B. Spörndly E. Reyes-Sánchez N. Salmerón-Miranda F. Halling M. (2013). Biomass production and chemical composition of *Moringa oleifera* under different planting densities and levels of nitrogen fertilization. *Agroforestry Systems* 87 81–92. 10.1007/s10457-012-9525-5

[B25] Miller G. L. (1959). Use of dinitrosalicylic acid reagent for determination of reducing sugar. *Anal. Chem.* 31 426–428. 10.1021/ac60147a030

[B26] Moyo B. Masika P. (2011). Nutritional characterization of Moringa (*Moringa oleifera* Lam.) leaves. *Afr. J. Biotechnol.* 10 12925–12933. 10.5897/ajb10.1599

[B27] Ndubuaku U. M. Nwankwo V. U. Baiyeri K. P. (2014). Influence of poultry manure application on the leaf amino acid profile, growth and yield of moringa (*Moringa oleifera* Lam.) *plants*. *Albanian J. Agric. Sci.* 13:42.

[B28] Ogbe A. O. Affiku J. P. (2011). Proximate study, mineral and anti-nutrient composition of *Moringa oleifera* leaves harvested from lafia, nigeria: potential benefits in poultry nutrition and health. *J. Microbiol. Biotechnol. Food Sci.* 1:296.

[B29] Olaofe O. Adeyeye E. I. Ojugbo S. (2013). Comparative study of proximate, amino acids and fatty acids of *Moringa oleifera* tree. *Elixir Appl. Chem.* 54 12543–12554.

[B30] Olukomaiya O. O. Adiamo O. Q. Fernando W. C. Mereddy R. Li X. Sultanbawa Y. (2020). Effect of solid-state fermentation on proximate composition, anti-nutritional factor, microbiological and functional properties of lupin flour. *Food Chem.* 315:126238. 10.1016/j.foodchem.2020.126238 32000081

[B31] Ovissipour M. Abedian A. Motamedzadegan A. Rasco B. Safari R. Shahiri H. (2009). The effect of enzymatic hydrolysis time and temperature on the properties of protein hydrolysates from *Persian sturgeon* (*Acipenser persicus*) viscera. *Food Chem.* 115 238–242. 10.1016/j.foodchem.2008.12.013

[B32] Phatsimo G. M. Cukrowska E. Chimuka L. (2015). Development of pressurized hot water extraction (PHWE) for essential compounds from *Moringa oleifera* extracts. *Food Chem.* 172 423–427. 10.1016/j.foodchem.2014.09.047 25442573

[B33] Popoola J. O. Obembe O. O. (2013). Local knowledge, use pattern and geographical distribution of *Moringa oleifera* Lam. (*Moringaceae*) in Nigeria. *J. Ethnopharmacol.* 150 682–691. 10.1016/j.jep.2013.09.043 24096203

[B34] Sanjukat S. Rai A. K. Muhammed A. Jeyaram K. Talukdar N. C. (2015). Enhancement of antioxidant properties of two soybean varieties of Sikkim Himalayan region by proteolytic Bacillus subtilis fermentation. *J. Funct. Foods.* 14 650–658. 10.1016/j.jff.2015.02.033

[B35] Sarmadi B. H. Ismail A. (2010). Antioxidative peptides from food proteins: a review. *Peptides* 31 1949–1956. 10.1016/j.peptides.2010.06.020 20600423

[B36] Sasipriya G. Siddhuraju P. (2012). Effect of different processing methods on antioxidant activity of underutilized legumes, *Entada scandens* seed kernel and *Canavalia gladiata* seeds. *Food Chem. Toxicol.* 50 2864–2872. 10.1016/j.fct.2012.05.048 22683485

[B37] Shi C. He J. Yu J. Yu B. Mao X. Zheng P. (2016). Physicochemical properties analysis and secretome of *Aspergillus* niger in fermented rapeseed meal. *PLoS One* 11:e0153230. 10.1371/journal.pone.0153230 27049858 PMC4822828

[B38] Shi H. H. Su B. Chen X. X. Pian R. Q. (2020). Solid state fermentation of *Moringa oleifera* leaf meal by mixed strains for the protein enrichment and the improvement of nutritional value. *PeerJ* 8:e10358. 10.7717/peerj.10358 33240663 PMC7680055

[B39] Singh B. Satyanarayana T. (2011). Microbial phytases in phosphorus acquisition and plant growth promotion. *Physiol. Mol. Biol. Plants* 17 93–103. 10.1007/s12298-011-0062-x 23572999 PMC3550544

[B40] Stevens C. G. Ugese F. D. Otitoju G. T. Baiyeri K. P. (2016). Proximate and anti-nutritional composition of leaves and seeds of *Moringa oleifera* in Nigeria: a comparative study. *Agro-Science* 14 9–17. 10.4314/as.v14i2.2

[B41] Teixeira E. M. B. Carvalho M. R. B. Neves V. A. Silva M. A. Arantes-Pereira L. (2014). Chemical characteristics and fractionation of proteins from *Moringa oleifera* Lam. leaves. *Food Chem.* 147 51–54. 10.1016/j.foodchem.2013.09.135 24206684

[B42] Tripathi M. K. Mishra A. S. (2007). Glucosinolates in animal nutrition: a review. *Anim. Feed Sci. Technol.* 132 1–27. 10.1016/j.anifeedsci.2006.03.003

[B43] Tufarelli V. Ragni M. Laudadio V. (2018). Feeding forage in poultry: a promising alternative for the future of production systems. *Agriculture* 8:81. 10.3390/agriculture8060081

[B44] Van Soest P. V. Robertson J. B. Lewis B. A. (1991). Methods for dietary fiber, neutral detergent fiber, and nonstarch polysaccharides in relation to animal nutrition. *J. Dairy Sci.* 74 3583–3597. 10.3168/jds.s0022-0302(91)78551-2 1660498

[B45] Vongsak B. Sithisarn P. Mangmool S. Thongpraditchote S. Wongkrajang Y. Gritsanapan W. (2013). Maximizing total phenolics, total flavaonoids contents and antioxidant activity of *Moringa oleifera* leaf extract by the appropriate extraction method. *Ind. Crops Prod.* 44 566–571. 10.1016/j.indcrop.2012.09.021

[B46] Wang C. Shi C. Su W. Jin M. Xu B. Hao L. (2020). Dynamics of the physicochemical characteristics, microbiota, and metabolic functions of soybean meal and corn mixed substrates during two-stage solid-state fermentation. *mSystems* 5 e501–e519. 10.1128/mSystems.00501-19 PMC7018524 32047057

[B47] Wang J. Cao F. Su E. Zhao L. Qin W. (2018a). Improvement of animal feed additives of ginkgo leaves through solid-state fermentation using *Aspergillus niger*. *Int. J. Biol. Sci.* 14:736. 10.7150/ijbs.24523 29910684 PMC6001676

[B48] Wang J. Cao F. Zhu Z. Zhang X. (2018b). Improvement of quality and digestibility of *Moringa oleifera* leaves feed via solid-state fermentation by *Aspergillus niger*. *Int. J. Chem. React. Eng.* 16:20180094.

[B49] Xie P. J. Huang L. X. Zhang C. H. Zhang Y. L. (2016). Nutrient assessment of olive leaf residues processed by solid-state fermentation as an innovative feedstuff additive. *J. Appl. Microbiol.* 121 28–40. 10.1111/jam.13131 26991541

[B50] Yusuf A. O. Mlambo V. Iposu S. O. (2018). A nutritional and economic evaluation of *Moringa oleifera* leaf meal as a dietary supplement in West African dwarf goats. *S. Afr. J. Anim. Sci.* 48 81–87. 10.4314/sajas.v48i1.10

[B51] Zeng B. Sun J. J. Chen T. Sun B. L. He Q. Chen X. Y. (2018). Effects of *Moringa oleifera* silage on milk yield, nutrient digestibility and serum biochemical indexes of lactating dairy cows. *J. Anim. Physiol. Anim. Nutr.* 102 75–81. 10.1111/jpn.12660 28299866

[B52] Zhang M. Huang Y. Zhao H. Wang T. Xie C. Zhang D. (2017). Solid-state fermentation of *Moringa oleifera* leaf meal using *Bacillus pumilus* CICC 10440. *J. Chem. Technol. Biotechnol.* 92 2083–2089.

[B53] Zuo S. S. Niu D. Z. Ning T. T. Zheng M. L. Jiang D. (2017). Protein enrichment of sweet potato beverage residues mixed with peanut shells by *Aspergillus oryzae* and *Bacillus subtilis* using central composite design. *Waste Biomass Valorization* 9 835–844. 10.1007/s12649-017-9844-x

[B54] Zuo Y. Chen H. (2002). Simultaneous determination of catechins, caffeine and gallic acids in green, Oolong, black and pu-erh teas using HPLC with a photodiode array detector. *Talanta* 57 307–316. 10.1016/s0039-9140(02)00030-9 18968631

